# Feasibility and Impact of a Combined Supervised Exercise and Nutritional-Behavioral Intervention following Bariatric Surgery: A Pilot Study

**DOI:** 10.1155/2015/693829

**Published:** 2015-06-23

**Authors:** Friedrich C. Jassil, Sean Manning, Neville Lewis, Siri Steinmo, Helen Kingett, Fiona Lough, Andrea B. F. Pucci, W. H. Cheung, Nicholas Finer, Judith Walker, Jaqueline Doyle, Rachel L. Batterham

**Affiliations:** ^1^Centre for Obesity Research, Rayne Institute, Department of Medicine, University College London, London WC1E 6JJ, UK; ^2^UCLH Centre for Weight Loss, Metabolic and Endocrine Surgery, University College London Hospitals, Ground Floor West Wing, 250 Euston Road, London NW1 2PG, UK; ^3^Cardiovascular Health, The Hatter Institute, 67 Chenies Mews, London WC1E 6HX, UK; ^4^National Institute of Health Research University College London Hospitals Biomedical Research Centre, London W1T 7DN, UK

## Abstract

*Background.* Lifestyle intervention programs after bariatric surgery have been suggested to maximise health outcomes. This pilot study aimed to investigate the feasibility and impact of an 8-week combined supervised exercise with nutritional-behavioral intervention following Roux-en-Y gastric bypass and sleeve gastrectomy. *Methods.* Eight female patients (44 ± 8 years old, BMI = 38.5 ± 7.2 kgm^−2^) completed the program. Before and after intervention, anthropometric measures, six-minute walk test (6MWT), physical activity level, eating behavior, and quality of life (QoL) were assessed. Percentage weight loss (%WL) outcomes were compared with a historical matched control group. *Results.* The program significantly improved functional capacity (mean increment in 6MWT was 127 ± 107 meters, *p* = 0.043), increased strenuous intensity exercise (44 ± 49 min/week, *p* = 0.043), increased consumption of fruits and vegetables (*p* = 0.034), reduced consumption of ready meals (*p* = 0.034), and improved “Change in Health” in QoL domain (*p* = 0.039). The intervention group exhibited greater %WL in the 3–12-month postsurgery period compared to historical controls, 12.2 ± 7.5% versus 5.1 ± 5.4%, respectively (*p* = 0.027). *Conclusions.* Lifestyle intervention program following bariatric surgery is feasible and resulted in several beneficial outcomes. A large randomised control trial is now warranted.

## 1. Introduction

Obesity is recognized as one of the major public health challenges of the 21st century. Currently, more than 2.1 billion people, approximately 30% of the global population, are overweight or obese. More worryingly, if the prevalence of obesity continues on its current trajectory, almost half of the world's adult population will be overweight or obese by 2030 [1]. To date, bariatric surgery is the most effective form of weight loss intervention for patients with severe obesity (body mass index [BMI] ≥ 35 kgm^−2^ with a comorbid condition that would be improved by weight loss, or BMI ≥ 40 kgm^−2^) [2]. Following bariatric surgery, significant amelioration in obesity-related comorbidities and mortality have been reported [2–5], which has led to a marked increase in the numbers of procedures undertaken worldwide [6].

Roux-en-Y gastric bypass (RYGB) and sleeve gastrectomy (SG) are now the two most commonly performed procedures globally [6]. Despite the overall beneficial effects of bariatric surgery, we [7] and others [8–10] have reported a marked postoperative variability in weight loss and resolution of comorbidities. Maximising the health benefits obtained from bariatric surgery is therefore a key priority. Several preoperative clinical factors such as BMI, sex, age, and type 2 diabetes (T2D) are suggested to impact upon weight loss outcomes [11–18]. However, we have recently shown that early, 3–6-month postoperative weight loss is the strongest predictor of maximal and 2-year postoperative weight loss response [7]. Hence, targeting patients with poor early weight loss following surgery with an early intensive postoperative lifestyle intervention and behavioral support could enhance weight loss outcomes [7].

Previous studies have demonstrated that postoperative supervised exercise training had no significant additional impact on a short-term weight loss outcome and was unable to prevent loss of lean muscle mass, a key determinant of energy balance [19–22]. However, exercise training has been shown to prevent loss of muscle strength and improve physical function, which are important components required to perform activities of daily living [19, 20, 22, 23]. Moreover, improvement in physical and cardiorespiratory fitness, reduction in diastolic blood pressure, and improvement in insulin sensitivity and glucose effectiveness are among the beneficial outcomes of postoperative exercise training [19–21, 23, 24].

Postoperative counselling on lifestyle modification that focuses on nutrition and physical activity should be offered as part of standard care [25, 26]. Moreover, providing early postoperative nutrition education and behavioral intervention has been shown to enhance weight loss after surgery [27, 28]. Current guidelines however do not consider whether any particular group of bariatric patients would benefit from extra attention and care following surgery [25, 26]. As poor early postoperative weight loss is a good predictor of poor long-term weight loss outcome [7, 29, 30], we hypothesised that focusing additional attention on “early poor responders” might help them to achieve greater health benefits from surgery. However, evidence to support the beneficial outcomes of postbariatric lifestyle intervention is still limited. Moreover, the feasibility of providing exercise program following bariatric surgery is still lacking and warrants additional investigation [19, 20].

Our pilot study aimed to evaluate the feasibility and impact of an 8-week combined exercise and nutritional-behavioral intervention commencing between 3 and 6 months after surgery in patients following SG and RYGB. We aimed to recruit patients with poor or intermediate early weight loss response and to assess the impact of our program on functional capacity, physical activity level (PAL), eating behavior, and quality of life (QoL). The impact of the program on percentage weight loss (%WL) was assessed at 1 year after surgery and compared to a group of matched historical surgical patients.

## 2. Materials and Methods

### 2.1. Study Design, Setting, and Participants

This prospective intervention study was conducted for an 8-week period. The study participants were recruited from patients who underwent bariatric surgery (either RYGB or SG) in University College London Hospitals (UCLH), National Health Service Foundation Trust, London, UK. The exercise intervention was delivered initially within a hospital gym facility. Female patients represent the majority of patients undergoing surgery at UCLH (approximately 70%); thus we restricted this pilot study to female patients [7, 11]. Inclusion criteria were female sex, ≥ 18 years of age, residency within a 25-mile radius of UCLH, 3-month postsurgery weight data available, and currently at 3–6 months after surgery. Exclusion criteria were medical contraindication such as cardiovascular disease, uncontrolled hypertension, and functional limitation. Twenty patients fulfilled our inclusion criteria.

**Figure 1:**
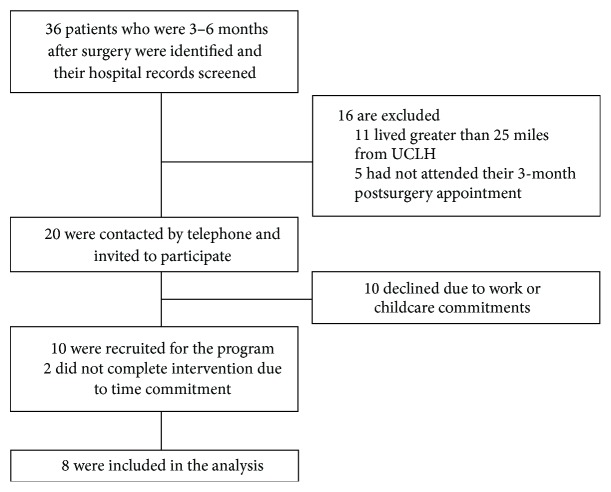
Flow of participant screening and recruitment.

Percentage weight loss (%WL) data were calculated using the following formula: %WL = [(weight on day of surgery − weight at time-point after surgery)/weight on day of surgery] × 100. Initially, patients with poor weight loss response, defined by weight loss of less than 15% at 3-month follow-up, were identified and invited by telephone to participate but subsequently patients with greater weight loss were invited. Ten patients agreed to participate; the remaining ten were unable to participate due to work or childcare commitments. However, 2 patients were unable to complete the intervention due to time commitment and therefore only 8 patients were included in the analysis (Figure 1).

### 2.2. Initial Assessment

A medical screening was carried out for all eligible patients prior to enrolment. This comprehensive assessment included review of patients' past medical history, in particular, their cardiac history, current medical state, medications, micronutrient supplement, and current PAL. All enrolled patients gave their informed consent prior to participation. Baseline anthropometric variables (height and weight), blood pressure, resting heart rate (HR), and self-reported eating behavior and QoL were documented. A food diary was administered to each patient to self-monitor their food intake in the week before the study commenced, which was later used by the dietitian and psychologist to guide the content of the nutritional-behavioral change sessions. A baseline six-minute walk test (6MWT) was undertaken.

### 2.3. Intervention

Each weekly session comprised a 60-minute exercise training period followed by a 60-minute group discussion on lifestyle education and nutritional-behavioral change sessions. The exercise program was designed, delivered, and supervised by a physiotherapist and an exercise specialist, based on frequency, intensity, type, and time (FITT) training principles. The exercise intensity varied according to individualized functional capacity and increased progressively every week according to the ease of performance. In each session, maximum exercise efforts of all patients were targeted at scale 13 “somewhat hard physical activity” based on Borg's Resting Perceived Exertion (RPE) scale [31].

The exercise program consisted of 15 minutes of warm-up and 30 minutes of workout period followed by 15 minutes of cool-down. The workout was based on a circuit design with interval approach comprised of six stations that combined arm and leg exercises performed using exercise equipment such as sand bags, step platform, resistance bands, treadmill, and stationary bicycle. Workout at each station was performed for three minutes with one-minute active recovery that involved toe and ankle pumping. Patients with knee and back problem performed seated exercises with extra supervision. The exercise intensity was increased every week by reducing recovery time at each station.

For exercise at home, patients were encouraged to undertake 30 minutes of moderate intensity exercise, combining cardiovascular and resistance training, 5 days a week. Patients were given a resistance band and a pedometer to self-monitor steps count. They were also advised to set individual weekly exercise goals and provided with home activity diary. Review of exercise goals including problems and barriers was undertaken at the following week. These were not subject to formal analysis. However, reported PAL were assessed at the end of the 8-week program and compared to baseline PAL. Upon completion of this 8-week program, patients were offered additional 8 weeks of exercise program in the local community gym monitored by an exercise specialist.

The lifestyle education and behavior change component was divided into two phases, conducted by a physiotherapist, an exercise specialist, a dietitian, and a psychologist. The first four weeks focused on exercise education tackling exercise variety, barriers, and dealing with back and joint problems. The following four weeks focused on nutritional behavior change, with emphasis on regular eating patterns, portion control, and balanced meals. Self-regulatory behavior change techniques from the Behavior Change Technique Taxonomy (self-monitoring using a food and exercise diary, barrier identification/problem solving, and weekly behavioral goal setting) underpinned both phases of the intervention [32]. All patients were taught the principles of SMART (specific, measurable, attainable, realistic, and timely) goal setting and encouraged to use this in relation to making changes to exercise and eating behavior throughout the course. All sessions were delivered in a group format, consisting of an educational component, followed by group discussion.

### 2.4. Six-Minute Walk Test (6MWT)

Patient's functional capacity was measured using 6MWT, conducted by an exercise specialist. The 6MWT is a self-paced, submaximal assessment of functional capacity used to prescribe appropriate exercise. It has been validated in obese population [33] and often used for bariatric patients [34, 35]. The test was performed for all patients according to the standard protocol in the “American Thoracic Society Statement: Guidelines for the Six-minute Walk Test” [36]. Briefly, patients were instructed to walk at their regular pace along an even 25 meters of undisturbed hospital corridor, marked every 5 meters. They were advised to cover as much distance as they could, walking back and forth for 6 minutes, monitored using a stopwatch. During the test, patients were allowed to slow down, stop and take a rest as necessary, but resume walking once they were ready. Minimal encouragement was given using the standard phrases in the guidelines and patients were asked to self-rate their level of exhaustion based on the “Talk Test,” Borg's RPE scale [31]. This is a simple method used to subjectively measure the intensity of PAL, rated from scale 6 = no exertion at all to scale 20 = maximal exertion. Perceived exertion is based on the person's experience of changes in their heart and breathing rate, sweating, and muscle fatigue when performing physical activity. This method was considered a good estimate of the actual HR during physical activity. The pre- and posttest HR, total distance covered, posttest Borg's RPE rating, number of stops, and any physical complaints were recorded.

### 2.5. Eating Behavior and Food Frequency Questionnaire

A questionnaire regarding eating behavior and food frequency was created and administered to patients. The eating behavior section of the questionnaire was focused on postbariatric dietary practice, which is a list of behaviors associated with better weight loss outcomes, such as regular eating, staged meal progression, eating slowly, not drinking during a meal, self-monitoring, and goal setting. Items also asked about patients' self-awareness of eating habits and knowledge of balanced meals and appropriate portion sizes.

The second section asked about food frequency and was used to evaluate the intake of calorie-dense foods such as take-away meals, fried foods, ready meals, and “soft calorie” food such as fizzy drinks, fruit juices, liquid meals, crisp, cakes, biscuits, chocolate, and sweets. Responses were scored by 0 = never, 1 = less than once a week, 2 = more than once a week, and 3 = almost every day. For intake of dietary fiber (fruits and vegetables), responses were scored by 0 = less than one portion daily, 1 = 1 to 2 portions daily, 2 = 3 to 4 portions daily, and 3 = 5 or more portions daily.

### 2.6. The Physical Fitness and Activity Questionnaire

The physical fitness and activity questionnaire was adapted from the National Audit of Cardiac Rehabilitation [37]. Patients reported their time spent on moderate and strenuous intensity physical activities in a week. The questionnaire also evaluates whether patients meet exercise recommendations for the general population.

### 2.7. The Dartmouth Cooperative Functional Assessment Charts (COOP)

The Dartmouth COOP is a simple, reliable, and quick self-administered questionnaire that is used to measure health-related quality of life (HRQL) [38]. Patients respond based on a scale ranging from 1 to 5 (1 = optimal) in 9 domains: Physical Fitness, Feelings, Daily Activities, Social Activities, Pain, Change in Health, Overall Health, Social Support, and QoL. A score of 1–3 is categorised as normal and 4-5 as abnormal.

### 2.8. Post-Intervention Assessment

The postintervention assessment was undertaken a month after completion of the program. This comprehensive assessment included review of patient's current medical state, medications, and current physical activity. Body weight, blood pressure, and resting HR were documented. Also, a repeat 6MWT was undertaken and the pre- and posttest HR, total distance covered, posttest Borg's RPE rating, number of stops, and any physical complaints were recorded.

### 2.9. Feasibility and Acceptability

The feasibility and acceptability of this lifestyle intervention was assessed based on the attrition rate. The program feedback was obtained from a questionnaire and by interview during the postintervention assessment.

### 2.10. Historical Control Patients and Impact on Postsurgery Weight Loss

The case-matched historical control group were identified from our electronic database of bariatric surgical patients with 3- and 12-month postsurgery weight data [7]. Two historical controls, matched for sex, surgical procedure, day of surgery BMI, rate of weight loss at 3-month visit (%WL per week), age, and T2D status, were identified for each intervention patient (Table 1). These patients were given standard postsurgical care involving dietetic follow-up at 3 and 9 months postoperatively and surgical follow-up at 6 months and annually thereafter. %WL and ΔBMI at 12 months after surgery were calculated. To further assess the impact of the intervention, %WL and ΔBMI for the 3–12-month postsurgery period were also calculated. The data from each patient and the averaged data from their two historical matched controls were compared (Table 2).

### 2.11. Statistical Analysis

Analysis was performed using Statistical Package for Social Study version 22 (SPSS v22). Descriptive statistics were used to explain study population at baseline. Unless otherwise stated, data were presented as mean and standard deviation (±SD) and percentage (%) for categorical data. Nonparametric tests were used to detect significant differences in postintervention functional capacity and PAL. McNemar and Wilcoxon signed-rank tests were used to analyze changes in eating behavior, food frequency, and QoL. Mann-Whitney *U* test was used to compare the impact of standard care or a combined supervised exercise with nutritional-behavioral intervention on weight loss outcomes at 1 year after surgery. A *p* value of <0.05 indicated the presence of a statistically significant difference.

## 3. Results

### 3.1. Study Participants

Eight patients completed the program. Six patients had undergone SG and two patients had undergone RYGB. The duration after surgery ranged from 3 to 6 months. The mean (±SD) age and BMI before intervention were 44 (±8) years and 38.5 (±7.2) kgm^−2^, respectively. The ethnicities of the patients were Caucasian (*n* = 4), Indian (*n* = 2), Caribbean (*n* = 1), and African (*n* = 1). Two patients had T2D, two had a diagnosis of polycystic ovarian syndrome, three had treated hypertension, two had controlled asthma, one had fibromyalgia, and one had treated hypothyroidism. The mean (±SD) total %WL at recruitment was 12.9 (±5.4) % and 19.6 (±8.5) % at the postintervention assessment.

The control group comprised 16 historical patients who had previously undergone bariatric surgery at UCLH (Table 1). There were no significant differences in characteristics between intervention and the control group (Table 3).

### 3.2. Feasibility and Acceptability

Two patients attended all the sessions and the other patients attended seven (*n* = 1), six (*n* = 1), five (*n* = 2), four (*n* = 1), and three sessions (*n* = 1), respectively. The median number of sessions attended was six. Patients who failed to come for the session without prior notice were contacted by a telephone call to promote compliance. The commonest reason reported for not attending was time constraints. No major health problems were reported.

Two patients were unable to attend the postintervention assessment due to personal reasons. Their postintervention weight was obtained via a phone call and during the 6-month dietetic follow-up. One patient who attended the postintervention assessment was unable to do the 6MWT as patient was feeling unwell due to cold/flu.

At the end of program assessment, all patients stated that they were satisfied with the coaching and exercise input provided by the physiotherapist and exercise specialist. Based on the attendance rate, once-per-week supervised exercise was deemed acceptable by all participants. With regard to nutritional-behavioral change sessions, two patients stated that they would have preferred a one-to-one session with the dietitian in order to obtain individualised feedback from their reported food diary. Overall, patients were satisfied with the program and suggested that the program should be offered to all bariatric patients.

### 3.3. Anthropometric Measures, PAL, and 6MWT Outcomes at the End of the Program

There were significant changes in body weight, BMI, and total %WL observed at approximately 14 weeks after the baseline assessment (*p* < 0.05) (Table 4). With regard to PAL outcome, none of the patients reported performing any form of strenuous intensity activity prior to intervention. However, after the program, the mean (±SD) time spent on strenuous activity was 44 (±49) minutes/week, (*p* < 0.05). No significant differences were reported for moderate physical activity. Overall, 37.5% of the patients reported that they had been able to fulfill the exercise recommendation for moderate and strenuous intensity per week, respectively, at the end of the program (Table 4).

**Figure 2:**
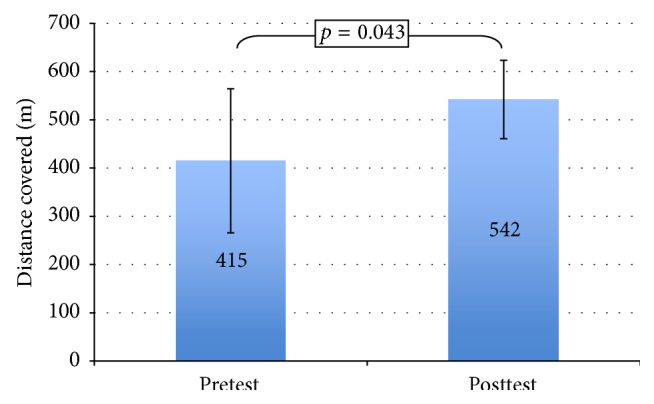
Changes in 6MWT before and after intervention (*n* = 5).

A significant increased distance covered for the 6MWT was observed in all patients (*n* = 5) who attended the postintervention 6MWT assessment with mean (±SD) increment of +127 (±107) meters (*p* < 0.05). Before the intervention, the baseline mean (±SD) of distance covered was 415 (±149) meters and this was significantly increased to 542 (±81) meters at the postintervention assessment. All patients improved their walking distance more than 25 meters from baseline (Figure 2).

### 3.4. Eating Behavior Outcomes

After intervention, all patients were able to tolerate a solid/normal diet. Patients reported increased awareness of the importance of routine breakfast, regular eating pattern, self-monitoring of food intake, and increased understanding of appropriate portion sizes and well-balanced meals. The proportion of patients with poor dietary habits (such as having meal and drinks together, grazing, self-starvation, late night eating habit, and eating leftover foods) decreased. No changes were seen in the practice of self-monitoring of body weight and ability to set and stick to SMART goals (Table 5).

Prior to the intervention, patients reported consuming “soft calorie” foods more than once a week; these included liquid meals, fruit juice, and sugary foods (cakes, biscuits, chocolate, and sweets). The frequency was reduced at postintervention assessment apart from fruit juice, although this did not reach statistical significance. The intake of fruits and vegetables increased significantly (*p* < 0.05) with median intake of 3 to 4 portions daily and a significant reduction (*p* < 0.05) was reported in the consumption of ready meals (Table 6).

### 3.5. QoL Outcomes

Prior to the intervention, the median score was normal for almost all domains in the Dartmouth COOP charts except for physical fitness with a median score of 3.5. Improvement in median score was observed in all domains with a significant improvement score in “Change in Health” domain at the end of program (*p* < 0.05) (Table 7).

### 3.6. Impact of the Program on Long-Term Weight Loss Outcomes

**Figure 3:**
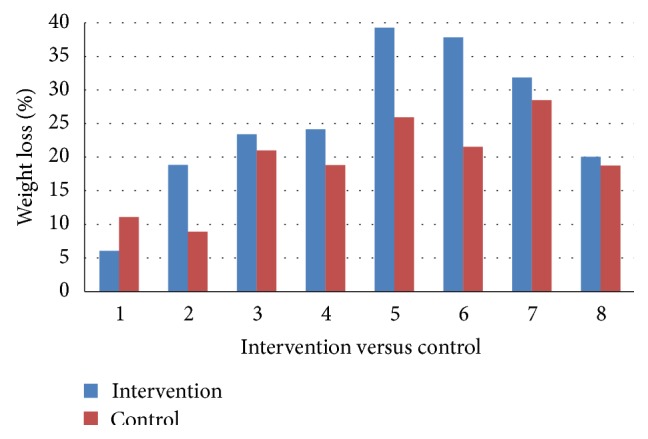
Comparison of %WL at 12-month postsurgery visit for each patient with the averaged data from their two case-matched controls.

**Figure 4:**
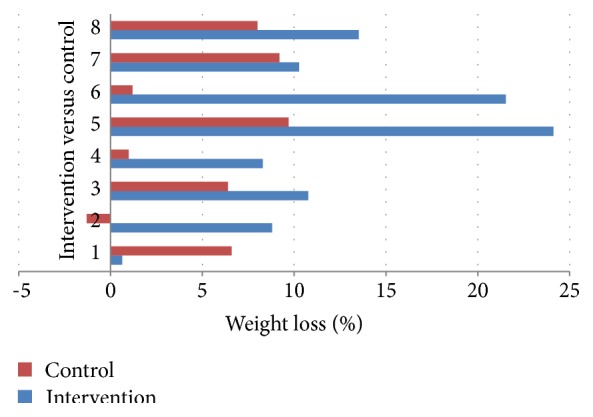
Comparison of %WL during 3–12-month postsurgery period for each patient with the averaged data from their two case-matched controls.

No significant differences were observed between the intervention and control group for %WL at 12 months' follow-up (Figure 3) or ΔBMI at 12 months after surgery (25.2 ± 11% versus 19.3 ± 7.2%, *p* = 0.208, and 11.2 ± 5 kgm^−2^ versus 8.7 ± 3.1 kgm^−2^, *p* = 0.189, resp.). However, %WL at 3–12-month period was significantly higher in the intervention group than the control (12.2 ± 7.5 versus 5.1 ± 5.4, *p* = 0.027) (Figure 4) with *p* = 0.05 for ΔBMI at 3–12-month period between the intervention and control group (5.5 ± 3.5 kgm^−2^ versus 2.4 ± 4.5 kgm^−2^).

## 4. Discussion

To our knowledge, this is the first study combining supervised exercise training with a nutritional-behavioral intervention given in a group setting for postoperative bariatric patients. Our findings show that a combined supervised exercise with nutritional-behavioral intervention program offered to postbariatric patients as early as 3 months after surgery is feasible and acceptable. 50% of patients attended between 6 and 8 sessions, with all patients being satisfied with the program provided. Importantly, this 8-week program significantly improved functional capacity, increased strenuous intensity exercise, increased consumption of fruits and vegetables, but reduced consumption of ready meals and improved “Change in Health” in QoL domain.

Only 50% of patients contacted were able to participate. Patients who were invited to participate were only given the option of attending a fixed session that was scheduled during working hours and in addition we required them to start within a 2-week period. These constraints are likely to have impacted upon patients' ability to attend. Hence, for future studies, participants should be recruited as early as feasible and offered a selection of time slots for the program. Problems scheduling the time needed for exercise training into daily routine were reported by participants to be the main reason for drop-out and inability to attend sessions. Similarly, problems allocating time have been reported in other studies that required patients to attend a 3-times-a-week program for a 12-week period [22–24]. This is in agreement with findings from a recent quantitative study involving 366 postsurgical patients which demonstrated that time (28.2%) was the most frequently reported barrier to exercise, followed by difficulties in maintaining exercise behavior (20.5%) and problems with pain and chronic illness (18.6%) [39]. In the present study, “health problems” was the second most reported reason for not being able to attend the scheduled exercise training; some patients reported back, knee, and joint pain that required an exercise regimen with less impact. Providing a suitable type of exercise for these patients is as important as negative beliefs about exercise, such as a high fear of injury, which, when present at 1 year after surgery, was strongly associated with less physical activity 2 years after surgery in one study [40].

According to King and Bond, physical activity counselling or exercise-related services should be provided following surgery and in view of physical, motivational, and external barriers faced by patients be tailored to individual needs and clinical status [41]. However, in a further study, patients reported lack of or inadequate advice regarding physical activity from their bariatric facility [39]. In our study, patients reported that the program provided helped them to improve their knowledge and motivation to exercise; hence they strongly suggested that this beneficial program be implemented on an ongoing basis.

A previous study has shown that walking limitation is common amongst obese individuals, even those with lower BMI range and younger age [42]. Improvement in impaired functional capacity prior to surgery as assessed by 6MWT, for example, has been observed as early as 7 to 12 months following bariatric surgery [35]. This is explained by the rapid weight loss that results from the bariatric procedure itself which makes walking activity less exhausting. Improved functional capacity can be accelerated by supervised exercise training [22–24], suggesting a role for enhancing fitness over and above weight loss. In the present study, we found that all patients attending for a repeat 6MWT improved their walking capacity. An increase of 25 meters in 6MWT is considered as clinically significant [43, 44], but this has not been validated in an obese population.

A recent systematic review and meta-analysis has emphasized the importance of exercise in the aftercare of bariatric surgery patients [45]. Based on this review, to be classified as an “exerciser,” a patient's PAL should be equal to or higher than the recommended level for general population (minimum of 30 minutes daily or equal to 150 minutes a week). In the present study, more patients reported to have met the “exerciser” criterion at the end of the program. According to Wouters et al., changes in PAL could be explained by a reduction in fear of injury and embarrassment, together with increased belief in the benefits of exercise after bariatric surgery [40].

The group exercise training that involved pairing of patients with similar physical conditions may also influence exercise motivation and help to develop a supportive environment. Moreover, the improvement of PAL might also be contributed by the effectiveness of group sessions, where the physiotherapist and exercise specialist emphasize the importance of setting realistic, measurable, and attainable short-term exercise goals in combination with self-monitoring exercise activity. This is an important strategy as many bariatric patients have problem achieving their physical activity goals in a week [46]. Moreover, although the effectiveness of self-monitoring physical activity in bariatric patients is unknown, higher PAL and weight loss were observed among nonsurgical obese patients who adhere well with a self-monitoring strategy [47].

It is known that adherence to postoperative dietary guidelines is crucial as it is associated with weight loss outcomes [48]. Based on the bariatric food pyramid, patients should avoid calorie-dense foods such as high saturated and trans fat, high sugar, and carbonated and alcoholic beverages whereas intake of fruits and vegetables should meet 2 to 3 servings daily [49]. However, a recent retrospective study analysing food intake based on bariatric food pyramid among 172 patients at 6 months after surgery found that half of the patients had insufficient fruit intake, while more than 80% did not consume vegetables as recommended [50]. In our study, patients reported intake of “soft calorie” or “meltable” foods more than once a week. Their intake of fruits and vegetables was only 1 to 2 portions daily, which was below the recommendation. However, substantial changes were observed after the intervention with significant reduction in the consumption of ready meals and a significant increase in fruits and vegetables that meets the recommendation. Indeed, as explained by Mathes and Spector, postsurgical food selection is influenced by nutritional counselling and the type of food advised following surgery [51].

Several habits toward weight loss strategy remain unchanged that includes self-monitoring of body weight and ability to set and stick to SMART goals despite nutritional-behavioral change counselling given by a dietitian and psychologist. In view of this, we proposed that individual dietary counselling should be given for these particular patients to promote better understanding.

Before the intervention, all patients had normal scores for 8 domains of the Dartmouth COOP charts apart from physical fitness. This may be due to the fact that this baseline measurement was undertaken at 3–6 months postoperatively rather than preoperatively and HRQL outcomes have been reported to improve early after surgery [52]. Following the intervention program, significant improvement was reported in “Change in Health” domain. This shows that even low volume of exercise as provided in the current study has helped patients to feel much better in their health. Consistent with previous studies, high-volume exercise among postbariatric patients was shown to improve physical function, self-esteem, sexual life, public distress, energy levels, and emotional and mental well-being as evaluated using the Short Form Health Survey (36) and Impact of Weight on QOL-Lite [21].

Previous studies have reported that supervised exercise training provided at earlier period after surgery did not contribute to additional weight loss outcome [19–22]. However, its long-term impact on weight loss has never been reported before. In the current study, we found that patients in the intervention group exhibit greater %WL during the 3–12-month postsurgery period compared to historical controls. This could have been facilitated by the beneficial short-term outcomes experienced by patients after the program.

This pilot study has several limitations. Firstly, this study only involved female patients. Previous studies have shown that there is a significant difference in terms of postsurgical weight loss outcome between genders [53]. Secondly, due to a very short notice and restrictive time flexibility for participants, we were only able to recruit a small sample size; thus our findings may be limited in their generalizability. Thirdly, we used a matched historical control group rather than a contemporaneous control group. Thus whilst we have weight data on this group we do not have comparable data for 6MWT, PAL, eating behavior, and QoL. Lastly, the Dartmouth COOP charts used to measure QoL have not yet been validated in an obese population hence the results should be interpreted with caution. A large prospective randomised control study is now warranted where patient are randomised to either standard care or an exercise and nutritional-behavioral intervention program with long-term data collection.

## 5. Conclusions

Our data suggest that a lifestyle intervention program that combined exercise training with nutritional-behavioral counselling given in group sessions is feasible and acceptable to postbariatric surgery patients. This program significantly improved functional capacity, increased strenuous intensity exercise, increased consumption of fruits and vegetables, but reduced consumption of ready meals and improved “Change in Health” in the QoL domain. These short-term beneficial outcomes have contributed to a greater weight loss at 3–12-month postsurgery period compared to historical controls receiving standard care. A large randomised control study is now warranted to expand upon our findings.

## Figures and Tables

**Table 1:** Baseline characteristics of the intervention and historical control groups (matched for procedure, age, day of surgery BMI, %WL/week at 3 months after surgery, and T2D status).

Intervention group						Control group							
Patient	Procedure	Age (y)	Day of surgery BMI (kgm^−2^)	%WL/week at 3 months after surgery	T2D status	Procedure	Age (y)		Day of surgery BMI (kgm^−2^)		%WL/week at 3 months after surgery		T2D status
								Ave		Ave		Ave	
1	SG	48	53	0.43	No	SG SG	52 56	54	59.0 49.6	54.3	0.22 0.48	0.35	No No

2	SG	54	34.5	0.73	Yes	SG SG	48 53	51	39.0 40.3	39.6	0.70 0.56	0.63	Yes Yes

3	RYGB	46	40.9	0.98	No	RYGB RYGB	46 41	44	42.3 42.2	42.3	1.03 0.78	0.91	No No

4	SG	40	54.1	1.13	No	SG SG	44 41	42	50.6 51.8	51.2	0.91 0.89	0.90	No No

5	RYGB	53	47.5	1.18	Yes	RYGB RYGB	49 62	55	46.9 45.5	46.2	1.16 1.19	1.17	Yes Yes

6	SG	29	42.6	1.19	No	SG SG	27 31	29	43.2 42.2	42.7	1.19 1.20	1.19	No No

7	SG	41	42.4	1.36	No	SG SG	50 40	45	42.4 42.5	42.5	1.31 1.26	1.28	No No

8	SG	46	46.5	0.50	No	SG SG	46 46	46	45.1 44.9	45.0	0.58 0.69	0.63	No No

Note: Ave: average; BMI: body mass index; %WL: percentage weight loss; RYGB: Roux-en-Y gastric bypass; SG: sleeve gastrectomy; T2D: type 2 diabetes.

**Table 2:** Comparison of weight loss outcome between the intervention and historical control groups.

Intervention group					Control group							
Patient	%WL at 12 months' follow-up	ΔBMI at 12 months after surgery (kgm^−2^)	%WL at 3–12-month period	ΔBMI at 3–12-month period (kgm^−2^)	%WL at 12 months' follow-up		ΔBMI at 12 months after surgery (kgm^−2^)		%WL at 3–12-month period		ΔBMI at 3–12-month period (kgm^−2^)	
						Ave		Ave		Ave		Ave
1	6.1	3.2	0.6	0.3	12.2 10	11.1	7.2 4.9	6.1	9.1 4.1	6.6	5.4 2.0	3.7

2	18.8	6.5	8.8	3.0	8.4 9.4	8.9	3.3 3.8	3.5	−2.5 −0.2	−1.3	−1.0 −0.1	−0.5

3	23.4	9.6	10.8	4.4	25.5 16.5	21	10.8 7.0	8.9	10.4 2.4	6.4	4.4 1.0	2.7

4	24.1	13.1	8.3	4.5	19.3 18.4	18.8	9.8 9.5	9.6	7.1 −5.1	1	3.6 −2.7	0.5

5	39.3	18.7	24.1	11.5	25.2 26.7	26	11.8 12.2	12.0	10.0 9.4	9.7	4.7 4.3	4.5

6	37.8	16.1	21.5	9.2	19.7 23.4	21.5	8.5 9.9	9.2	0.3 2.1	1.2	0.2 0.9	0.5

7	31.8	13.5	10.3	4.4	35.8 21.1	28.5	15.2 9.0	12.1	14.5 3.9	9.2	6.2 1.6	3.9

8	20.1	9.3	13.5	6.3	17.5 20	18.8	7.9 9.0	8.4	5.8 10.2	8.0	2.6 4.6	3.6

Note: Ave: average; BMI: body mass index; %WL: percentage weight loss.

**Table 3:** Comparison of baseline characteristics between intervention and historical control group.

Characteristic	Intervention (*n* = 8)	Control (*n* = 16)	*p* value
	Mean (±SD)		
Age (y)	44.8 (8.2)	45.5 (8.3)	0.599
Day of surgery BMI (kgm^−2^)	45.2 (6.5)	44.3 (5.2)	1.0
%WL at 3 months' follow-up	12.9 (5.4)	14.2 (5.7)	0.563

Note: BMI: body mass index; %WL: percentage weight loss.

**Table 4:** Anthropometry outcomes and changes in PAL.

Variable	Before	After	*p* value
	Mean (±SD)		
Weight (kg)	98 (20.1)	92.8 (20.4)	0.012
BMI (kgm^−2^)	38.5 (7.2)	36.5 (7.6)	0.012
BMI loss after surgery (kgm^−2^)	6.7 (3.1)	8.7 (3.8)	0.012
Total percentage weight loss (%)	12.9 (5.4)	19.6 (8.5)	0.012
PAL (min/week)			
Strenuous	0	44 (49)	0.043
Moderate	231 (272)	109 (66)	0.310

	*n* (%)		

Exercise recommendation (%)			
150 min/week of moderate intensity	2 (25)	3 (37.5)	1.0
75 min/week of strenuous intensity	0	3 (37.5)	0.125

Note: BMI: body mass index; PAL: physical activity level.

**Table 5:** Changes in eating behavior.

Eating behavior	Before		After		% change
	*n*	%	*n*	%	(*p*-value)
Regular breakfast	2	25	3	37.5	+12.5 (0.5)
Regular eating pattern	5	62.5	7	87.5	+25 (1.0)
Snacking	8	100	7	87.5	−12.5 (1.0)
Grazing	3	37.5	0	0	−33.3 (0.25)
Self-starvation	2	33.3	1	12.5	−20.8 (0.25)
Eating 1/4 of daily calories after evening meal	1	12.5	4	50	+37.5 (0.5)
Nocturnal eating	3	37.5	1	12.5	−25 (0.5)
Tolerating solid food	7	87.5	8	100	+12.5 (1.0)
Spending 15 minutes every meal	6	75	5	62.5	−12.5 (1.0)
Returning to leftover	5	62.5	3	37.5	−25 (1.0)
Drinking with meal	3	37.5	2	25	−12.5 (1.0)
Ability to set and stick to SMART goals	6	75	6	75	0 (1.0)
Awareness of proper eating behaviors	8	100	8	100	0 (1.0)
Self-monitoring food intake	2	33.3	8	100	+66.7 (0.13)
Self-monitoring body weight	4	50	4	50	0 (1.0)
Eating balanced meal	4	50	7	87.5	+37.5 (0.38)
Knowing the right portion size for each food group	6	75	8	100	+25 (0.5)

Note: SMART: specific, measurable, attainable, realistic, and timely.

**Table 6:** Changes in intake of calorie-dense foods, “soft calorie” foods, fruits, and vegetables.

Food frequency	Before		After		*p* value
	Median	IQR	Median	IQR	
Deep fried foods	1	0-1	1	1–1.25	0.317
Ready meals	1	1–2.25	0.5	0-1	0.034
Take-away meals	1	0–1.25	1	0.75–1	0.655
Crisp	1	0–2	0.5	0–1.25	0.083
Cakes, biscuits, chocolate, and sweets	2	1-2	1	1–1.25	0.083
Fizzy drinks	0	0–0.25	0	0-1	1.0
Fruit juice	2	1-2	2	0.75–2	0.257
Liquid meals	2	1.5–2	1	0.75–1	0.121
Fruits and vegetables	1	0.75–2	2	1.75–2.25	0.034

**Table 7:** Median (IQR) score for the Dartmouth COOP charts before and after intervention.

The Dartmouth COOP charts	Before		After		*p* value
	Median	IQR	Median	IQR	
*Domain *					
Physical Fitness	3.5	3-4	2	2–2.5	0.114
Feelings	2.5	2–3.5	2.5	1.75–3	0.236
Daily Activities	2	1–3	1.5	1-2	0.595
Social Activities	1	1-1	1	1-1	0.655
Pain	3	1.75–3	2.5	1.75–3.25	0.891
Change in Health	3	2.75–3	1.5	1–2.25	0.039
Overall Health	3	2.75–3.25	2.5	2-3	0.257
Social Support	3	2-3	2	2–3.5	0.705
Quality of Life	2.5	2-3	2	2-3	1.00
